# Identifying and addressing patient substance use: a survey of chiropractic clinicians

**DOI:** 10.1186/s12998-023-00490-4

**Published:** 2023-07-03

**Authors:** Jordan A. Gliedt, Maureen Reynolds, Steffany Moonaz, Cynthia R. Long, Robb Russell, Michael J. Schneider

**Affiliations:** 1 Department of Neurosurgery, Medical College of Wisconsin, 1155 N. Mayfair Rd, Milwaukee, WI 53226 USA; 2 School of Pharmacy, University of Pittsburgh, Pittsburgh, PA USA; 3 Southern California University of Health Sciences, Whittier, CA USA; 4 Maryland University of Integrative Health, Laurel, MD USA; 5 Palmer Center for Chiropractic Research, Palmer College of Chiropractic, Davenport, IA USA; 6 Department of Physical Therapy, University of Pittsburgh, Pittsburgh, PA USA; 7 Clinical Translation and Science Institute, University of Pittsburgh, Pittsburgh, PA USA

**Keywords:** Chiropractic, Alcohol use, Substance use, Opioid use, Prescription drug misuse, Surveys, Cross-sectional studies

## Abstract

**Background:**

Chiropractors commonly encounter patients who present for spine pain with parallel substance use. There is currently no widespread training within the chiropractic profession to prepare chiropractors to recognize and address substance use in clinical practice. The purpose of this study was to examine chiropractors’ confidence, self-perceptions, and interest in education associated with identifying and addressing patient substance use.

**Methods:**

A 10-item survey was developed by the authors. The survey addressed chiropractors’ assessment of their training, experiences, and educational interest/needs regarding identifying and addressing patient substance use. The survey instrument was uploaded to Qualtrics and was electronically distributed to chiropractic clinicians at active and accredited English-speaking Doctor of Chiropractic degree programs (DCPs) in the United States.

**Results:**

A total of 175 individual survey responses were returned from a total of 276 eligible participants (63.4% response rate) from 16 out of 18 active and accredited English-speaking DCPs (88.8% of DCPs) in the United States. Nearly half of respondents strongly disagreed or disagreed (n = 77, 44.0%) that they were confident in their ability to identify patients who misuse prescription medication. The majority of respondents (n = 122, 69.7%) indicated that they did not have an established referral relationship with local clinical providers who provide treatment for individuals who use drugs or misuse alcohol or prescription medications. Most respondents strongly agreed or agreed (n = 157, 89.7%) that they would benefit from participating in a continuing education course on topics related to patients who use drugs or misuse alcohol or prescription medications.

**Conclusions:**

Chiropractors indicated a need for training to help them identify and address patient substance use. There is a demand among chiropractors to develop clinical care pathways for chiropractic referrals and collaboration with health care professionals who provide treatment for individuals who use drugs or misuse alcohol or prescription medications.

## Introduction

Chiropractors are commonly utilized health care professionals in the United States. It is estimated that approximately 100 million chiropractic visits are completed annually [1]. In the United States, chiropractors predominantly provide portal-of-entry level care for spine pain, including chronic pain [2–7]. Along with primary care practitioners, chiropractors are the most frequently consulted first-contact health care professional for spine pain [4–7]. Though chiropractors primarily concentrate in the management of spine related disorders, chiropractors are ideally suited to recognize and facilitate referral for other health conditions due to their holistic approach that typically involves multiple visits over a course of care [8].

Substance use and substance use disorders are widespread in the United States [9–11]. There were more than 100,000 overdose deaths attributed to opioids and other drugs in the United States in 2021 [9, 10]. Alcohol related deaths are the third leading cause of preventable deaths in the United States [11]. There is a known relationship between pain and substance use [12–16]. Greater than 50% of regular opioid users describe back pain [17]. A systematic review by Corcoran, et al. revealed between 12 and 57% of chiropractic patients have parallel opioid medication prescription from a prescribing health care professional [18]. Further, a secondary analysis of National Health Interview Survey data by Ndetan, et al. indicated that approximately 1 out of 10 chiropractic patients consume hazardous levels of alcohol [19]. It is likely, therefore, that individuals seek chiropractic care for spine pain with concurrent substance misuse and substance use disorders.

Despite the prevalence of individuals presenting to chiropractors with concomitant pain and substance use, there is no consistent training within the chiropractic profession to prepare chiropractors to recognize and address substance use in clinical practice. Thus, it is our hypothesis that chiropractors lack tools to identify and address patient substance use and that there is a need for chiropractic continuing education in this field. The purpose of this study was to examine chiropractors’ confidence, self-perceptions, and interest in education associated with identifying and addressing patient substance use.

## Methods

### Study design

This was a cross-sectional survey of chiropractic clinicians who are involved in direct patient care at Doctor of Chiropractic degree programs (DCPs) in the United States between January 31, 2023 and March 31, 2023.

### Survey development

Informed by survey items from the Alcohol and Alcohol Problems Perceptions Questionnaire (AAPPQ) and the Drug and Drug Problems Perceptions Questionnaire (DDPPQ), the survey instrument used in this study was developed by the authors. The AAPPQ and the DDPPQ are valid and reliable tools commonly used to assess health care professionals’ attitudes toward the care of patients living with alcohol or drug use [20, 21]. We adopted concepts from survey items in the AAPPQ and the DDPPQ and included a separate concept of perceptions of training in substance use to develop a survey instrument specifically for chiropractors.

The survey instrument included a total of 10-items intended to understand chiropractors’ confidence, self-perceptions, and interest in education in methods of recognizing and addressing patient substance use. Examples of items included were “I am confident in my ability to identify patients who misuse prescription medication” and “I would benefit from participating in a continuing education course on the topics of patients who use drugs or misuse alcohol or prescription medications” (see Table 1 for all survey items). Responses were recorded on a 4-point Likert-type scale, ranging from strongly agree to strongly disagree. Demographic information was not collected with the survey instrument as it was deemed that demographics would not be contributory to the purpose of this study and would potentially make it possible to identify respondents for DCPs with a small number of chiropractic clinicians.

**Table 1:** Frequency of responses to survey items (n = 175)

Survey Item	Strongly Agree n (%)	Agree n (%)	Disagree n (%)	Strongly Disagree n (%)
I am confident that I can distinguish the differences between a patient who uses alcohol/prescription medication and a patient who misuses alcohol/prescription medication.	*22* (12.5)	*98* (56.0)	*50* (28.5)	*5* (2.8)
I am confident in my ability to identify patients who misuse alcohol.	*29* (16.5)	*92* (52.5)	*51* (29.1)	*3* (1.7)
I am confident in my ability to identify patients who misuse prescription medication.	*14* (8.0)	*84* (48.0)	*73* (41.7)	*4* (2.2)
I feel that it is important for chiropractors to routinely screen for patients who use drugs/alcohol/prescription medication, and to identify patients who misuse alcohol/prescription medication.	*70* (40.0)	*77* (44.0)	*21* (12.0)	*7* (4.0)
I have an established referral relationship with local clinical providers who treat patients who use drugs or misuse alcohol or prescription medications.	*20* (11.4)	*33* (18.8)	*80* (45.7)	*42* (24.0)
I am comfortable having conversations with my patients about drug/alcohol/prescription medication use and misuse.	*46* (26.2)	*79* (45.1)	*43* (24.5)	*7* (4.0)
Referral for treatment for a patient who uses drugs or misuses alcohol or prescription medications is not the responsibility of a chiropractor.	*5* (2.8)	*13* (7.4)	*78* (44.5)	*79* (45.1)
I had adequate training in chiropractic school about how to recognize and address patients who use drugs or misuse alcohol or prescription medications.	*3* (1.7)	*24* (13.7)	*85* (48.5)	*63* (36.0)
I am interested in learning more about communication skills that could enhance my discussions with patients about drug/alcohol/prescription medication use and misuse.	*68* (38.8)	*84* (48.0)	*19* (10.8)	*4* (2.2)
I would benefit from participating in a continuing education course on the topics of patients who use drugs or misuse alcohol or prescription medications.	*78* (44.5)	*79* (45.1)	*16* (9.1)	*2* (1.1)

### Population sampling

The target population for this study was chiropractic clinicians at all active and accredited English-speaking DCPs in the United States. The rationale for targeting this population was twofold. First, given that the clinical training provided at DCPs influences students’ future practice behaviors, [22] this study focused on chiropractors in clinical practice within DCP settings. Second, concentrating on chiropractors in clinical practice within DCPs allowed us to preliminarily explore the topic of patient substance use in chiropractic clinical practice with a wide geographic reach.

We defined eligible participants as individuals who were actively employed by an active and accredited English-speaking chiropractic college in the United States as an active chiropractic clinician who is engaged in direct patient care and/or providing direct oversight of patient care delivered by chiropractic students within their respective DCPs during the time of this study. Unpaid faculty in chiropractic practice external to a DCP-associated clinic with whom chiropractic students rotate with for community-based internships were not eligible for this study. We identified DCPs accredited by the Council on Chiropractic Education [23].

Established contacts known to the authors at 18 DCPs in the United States were identified based on their position and scope of work at their respective DCP (e.g., “Dean of Clinics”, “Director of Research”, etc.). An initial email was sent to each contact. This initial email contained a description of this research study and a request to forward a subsequent email with an invitation to participate in this study to all eligible participants at their respective DCPs. If contacts did not respond to the initial email, up to 3 follow up emails were sent to that contact. If the contact was unresponsive after the third follow up email, no additional attempts were made.

A response from contacts at 16 out of 18 of the identified accredited DCPs (88.8% of DCPs) in the United States was secured. A total of 276 individuals were identified as eligible participants from these 16 DCPs. A description of the individual and total sample sizes of eligible participants at each of the responding 16 DCPs is shown in Table 2.

**Table 2:** Chiropractic clinicians practicing at active and accredited English-speaking Doctor of Chiropractic degree programs in the United States

Doctor of Chiropractic Degree Programs	Location(s)	Chiropractic Clinicians
Cleveland University	Overland Park, Kansas	n = 18
Keiser University	West Palm Beach, Florida	n = 4
Life Chiropractic College West	Hayward, California	n = 26
Life University	Marietta, Georgia	n = 17
Logan University	Chesterfield, Missouri	n = 23
National University of Health Sciences (2 campus locations)	Lombard, Illinois	n = 5
	Seminole, Florida	n = 3
Northeastern College of Health Sciences	Seneca Falls, New York	n = 21
Northwestern Health Sciences University	Bloomington, Minnesota	n = 19
Palmer College of Chiropractic (2 campus locations)	Davenport, Iowa	n = 22
	Port Orange, Florida	n = 30
Parker University	Dallas, Texas	n = 18
Southern California University of Health Sciences	Whittier, California	n = 43
Texas Chiropractic College	Pasadena, Texas	n = 5
Western States University	Portland, Oregon	n = 12
University of Bridgeport	Bridgeport, Connecticut	n = 10
***Total Chiropractic Clinicians at Doctor of Chiropractic Degree Programs***		n = 276

*Note: The Palmer College of Chiropractic West DCP was excluded and was not contacted (soon to be inactive); The Universidad Central Del Caribe DCP was excluded (Spanish-speaking); Attempts to contact the D’Youville University and Sherman College of Chiropractic DCPs were unsuccessful

#### Survey administration

The survey instrument was uploaded to Qualtrics, an online survey platform [24]. The online survey was hosted by the Medical College of Wisconsin.

Initial individual emails were sent to the established contacts at each of the responding DCPs in this study. Reminder emails were sent to each DCP contact weekly with requests for the reminder emails to be forwarded to eligible participants at their respective DCP. The survey was closed on March 31, 2023.

### Ethics

This study was approved as an exempt educational survey by the Medical College of Wisconsin Institutional Review Board (IRB) – (IRB ID: PRO00046243). Prior to completion of the online survey, participants were provided with an online informational letter that notified them about the nature of this research study, the voluntary nature of participation, and that all responses were anonymous.

### Data management

Responses were automatically uploaded to the Qualtrics platform which is stored on a protected electronic database at the Medical College of Wisconsin.

### Data analysis

Descriptive statistics were obtained from Qualtrics. Frequency of responses to each of the survey items was calculated on the Qualtrics platform.

## Results

A total of 175 survey responses were returned from a total of 276 eligible participants (63.4% response rate) from 16 out of 18 DCPs (88.8% of DCPs) in the United States. Frequency of responses to each of the survey items is presented in Table 1. The majority of respondents strongly agreed or agreed (n = 120, 68.5%) that they were confident that they could distinguish the difference between a patient who uses alcohol/prescription medication and a patient who misuses alcohol/prescription medication. Consistent with this response, the majority of respondents strongly agreed or agreed (n = 121, 69.1%) that they were confident in their ability to identify patients who misuse alcohol. However, nearly half of the respondents strongly disagreed or disagreed (n = 77, 44.0%) that they were confident in their ability to identify patients who misuse prescription medication.

Most respondents strongly agreed or agreed (n = 147, 84.0%) that it is important for chiropractors to routinely screen for patients who use drugs/alcohol/prescription medication, and to identify patients who misuse alcohol/prescription medication. Most respondents strongly agreed or agreed (n = 125, 71.4%) that they are comfortable having conversations with patients about drug/alcohol/prescription medication use and misuse. In addition, most respondents (n = 157, 89.7%) indicated that referral for treatment for a patient who uses drugs or misuses alcohol or prescription medications is the responsibility of a chiropractor. However, the majority of respondents (n = 122, 69.7%) indicated that they did not have an established referral relationship with local clinical providers who provide treatment for individuals who use drugs or misuse alcohol or prescription medications.

Most respondents strongly disagreed or disagreed (n = 148, 84.5%) that they had adequate training in chiropractic school about how to recognize and address patients who use drugs or misuse alcohol or prescription medications. Most respondents (n = 152, 86.8%) indicated an interest in learning more about enhancing their communication skills to facilitate discussions with patients about drug/alcohol/prescription medication use and misuse. In addition, most respondents strongly agreed or agreed (n = 157, 89.7%) that they would benefit from participating in a continuing education course on topics related to patients who use drugs or misuse alcohol or prescription medications.

## Discussion

This is the first known study to assess chiropractic clinicians’ confidence, self-perceptions, and interest in education associated with identifying and addressing patient substance use. This study included practicing chiropractors within DCP clinic settings across the United States, including responses from participants from 16 out of 18 DCPs. Survey responses in this study suggest that there is a recognized need and an interest among chiropractors to participate in continuing education courses on the topic of patient substance use. Further, responses from this study suggest a need to develop and strengthen clinical pathways for chiropractic referrals and collaboration with health care professionals who provide treatment for individuals who use drugs or misuse alcohol or prescription medications.

Substance misuse is a major public health concern in the United States. The United States Preventive Services Task Force advises that health care professionals in portal-of-entry (primary care) settings – such as chiropractors in the United States – screen for substance use including non-medical use of prescription medications and alcohol [25]. Chiropractors are outstanding candidates to recognize individuals at the intersection of pain and substance use disorders. Given the link between substance use and back pain, and the regularity of substance use among chiropractic patients, chiropractors are very well positioned to play a sentinel role in identifying substance misuse and initiating treatment when appropriate. However, results of this study suggest that training is needed to properly equip chiropractors to consistently recognize and address patients presenting with spine complaints and concomitant substance use.

Evidence-based training programs in Screening, Brief Intervention, and Referral to Treatment (SBIRT), could be implemented in the chiropractic profession. Partnering with substance use experts who have successfully developed and implemented trainings in other allied health professions could be beneficial in expediently transferring substance use knowledge and skills to practicing chiropractors. For example, SBIRT trainings have been embedded into educational curriculum of several health care professions, including physician assistants, nurses, occupational therapists, physical therapists, psychologists, social workers, and pharmacists [26–28]. These trainings have been shown to be feasible and result in improved knowledge, attitudes, and perceived competency among learners to address substance use with their patients [26, 28].

Findings from this study also suggest that work is needed to initiate and support clinical pathways for chiropractic referrals and collaboration with health care professionals specializing in substance use. Approximately 70% of respondents of this study indicated that they did not have an established referral relationship with local clinical providers who provide treatment for patients who use drugs or misuse alcohol or prescription medications. This lack of interprofessional collaboration could have obvious implications on the chiropractic patient experience, such as delay in treatment for substance use disorders.

It is essential that chiropractors not only understand the indication for referral to treatment for substance use disorders, but also have a working referral relationship to effectively secure specialized treatment and services for those patients in need. Particularly given that chiropractors primarily work in isolated private practice settings, [29] developing interdisciplinary networking panels to promote integration and shared resources between substance use/addiction treatment professionals and chiropractors at the local, regional, and national levels is needed. Further, our findings highlight the important role of chiropractic educators and the need for an infusion of training in substance misuse within chiropractic college curricula.

### Limitations

This study has some limitations. First, this study is limited by the lack of psychometric analyses performed on our survey instrument. Therefore, the validity of the survey instrument may be compromised. For example, potential interpretation bias of individual survey items may impact our findings. Second, this survey study was conducted only among chiropractic clinicians at active and accredited English-speaking DCPs and may not be generalizable to practicing field chiropractors. However, given the impact of training on students’ practice habits, we hypothesize there is likely an association between DCP clinicians and chiropractic clinicians in the field, which could be examined in future research. Lastly, though locations of Internet Protocol (IP) addresses for individuals who completed the online survey were recorded, the online survey did not have a mechanism to ensure that each unique respondent could only complete the survey once.

## Conclusions

Chiropractors are widely used health care professionals in the United States. Chiropractors encounter patients who experience pain with concurrent substance use. This cross-sectional survey of practicing chiropractors in DCP clinic settings across the United States suggests there is a need and interest in participating in continuing education courses on identifying and addressing patient substance use. Findings also suggest that there is a need to develop and strengthen clinical care pathways for chiropractic referrals and collaborative care with health care professionals who provide treatment for individuals who use drugs or misuse alcohol or prescription medications.

## Data Availability

The dataset used and analyzed during the current study are available from the corresponding author upon reasonable request.

## List of abbreviations

DCP: Doctor of Chiropractic Degree Program
IRB: Institutional Review Board
SBIRT: Screening, Brief Intervention, and Referral to Treatment
IP: Internet Protocol
